# Synaptic κ‐Ga_2_O_3_ Photodetectors for Privacy‐Enhancing Neuromorphic Computing

**DOI:** 10.1002/advs.75160

**Published:** 2026-04-03

**Authors:** Yanqing Jia, Heming Lin, Hongliang Chang, Wenqing Niu, Yue Wang, Hang Lu, Abdullah AlQuwayzani, Yara Banda, Long Chen, Qingxiao Wang, Bambar Davaasuren, Mohamed Ben Hassine, Tien Khee Ng, Boon S. Ooi

**Affiliations:** ^1^ Photonics Laboratory, Electrical and Computer Engineering Division of Computer, Electrical and Mathematical Sciences and Engineering King Abdullah University of Science and Technology (KAUST) Thuwal Saudi Arabia; ^2^ Imaging and Characterization Core Lab King Abdullah University of Science and Technology (KAUST) Thuwal Saudi Arabia; ^3^ Electrical, Computer and Systems Engineering Rensselaer Polytechnic Institute Troy New York USA

**Keywords:** hardware‐level authentication, κ‐Ga_2_O_3_, neuromorphic inference capability, neuromorphic photodetector

## Abstract

Optoelectronic devices that unify sensing, memory, and computation offer a promising route toward intelligent and data‐local edge systems. Here, a multifunctional metal‐semiconductor‐metal neuromorphic photodetector based on the persistent photoconductivity (PPC) of κ‐phase gallium oxide (κ‐Ga_2_O_3_) is reported, enabling in‐sensor information processing and long‐term state retention within a single device element. Owing to pronounced PPC effect, nominally identical devices exhibit reproducible yet device‐distinguishable temporal photocurrent responses. These responses are exploited for hardware‐level authentication using a hybrid 1D deep embedding network, which achieves robust cross‐cycle verification performance with an Area Under the Curve (AUC) of about 0.97 and an Equal Error Rate (EER) of about 9%. Beyond authentication, the neuromorphic inference capability of the devices is evaluated using a hardware‐aware simulation framework, in which experimentally extracted conductance states are mapped to a quantization‐aware trained (QAT) artificial neural network (ANN) with 16 discrete levels. The quantized network achieves 98.17% accuracy and is subsequently converted into a leaky integrate‐and‐fire (LIF) spiking neural network (SNN), retaining 96.80% accuracy under device‐constrained operation. By performing sensing, authentication, and inference at device level, the κ‐Ga_2_O_3_ synaptic photodetectors establish a materials‐enabled pathway toward compact, intelligent, and privacy‐enhancing optoelectronic hardware for next‐generation edge systems.

## Graphical abstract

We report on a single‐element neuromorphic sensor based on the persistent photoconductivity (PPC) of κ‐phase Ga_2_O_3_ capable of sensing ultraviolet light and harnessing intrinsic data privacy. The approach establishes a materials‐enabled pathway toward compact, intelligent, and privacy‐enhancing optoelectronic hardware for next‐generation edge systems.

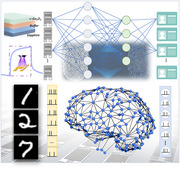

## 1 Introduction

The rapid proliferation of the Internet of Things (IoT) is driving the deployment of vast numbers of distributed sensing nodes across application domains ranging from smart infrastructure and industrial automation to environmental monitoring and healthcare. As the volume and dimensionality of data generated at the network edge continue to increase, conventional cloud‐centric computing architectures face growing challenges related to latency, energy consumption, and data privacy. In response, edge computing has emerged as a critical paradigm that seeks to process and interpret data as close as possible to the point of acquisition, thereby reducing communication overhead while enabling faster, more context‐aware responses. This shift places new and stringent demands on edge hardware, which must increasingly integrate sensing, memory, computation, and security within compact and energy‐efficient platforms [1, 2, 3].

A key challenge in realizing such intelligent edge systems lies in the fragmentation of functionality in conventional hardware architectures. Sensing, data storage, computation, and device authentication are typically implemented using physically and functionally distinct components, leading to increased system complexity, power consumption, and vulnerability to physical and cyber‐attacks. Moreover, security and privacy in many edge devices rely heavily on software‐based cryptographic protocols, which may be difficult to scale across massive device populations and can be compromised if the underlying hardware is accessed or cloned [4, 5]. These limitations motivate the exploration of alternative device concepts in which multiple functionalities are intrinsically co‐localized at the hardware level, enabling data‐local processing and hardware‐rooted authentication.

Neuromorphic electronic and optoelectronic devices provide a compelling framework for addressing these challenges. Inspired by the structure and function of biological neurons and synapses, neuromorphic devices are capable of mimicking time‐dependent, nonlinear behaviors such as short‐term facilitation, long‐term potentiation, and spike‐rate‐dependent plasticity [6, 7, 8, 9, 10, 11, 12, 13]. These properties enable information to be encoded, stored, and processed directly within the physical state of the device, supporting in‐memory and event‐driven computation with reduced energy overhead. In particular, optoelectronic synapses, which combine optical sensing with synaptic plasticity, have attracted increasing attention as candidates for intelligent sensors capable of perceiving and processing optical stimuli without extensive peripheral circuitry. Such devices are especially attractive for edge applications, where optical signals often serve as the primary information carriers.

In this context, wide‐bandgap and ultra‐wide‐bandgap semiconductors offer unique opportunities for neuromorphic optoelectronics. Among them, κ‐Ga_2_O_3_ has emerged as a promising material platform due to its large bandgap, intrinsic solar‐blind ultraviolet (UV) sensitivity, and compatibility with scalable thin‐film growth techniques [14, 15, 16, 17, 18, 19, 20]. Beyond these attributes, κ‐Ga_2_O_3_ exhibits pronounced persistent photoconductivity (PPC), arising from defect‐mediated carrier trapping and release processes. This long‐lived photoconductive response naturally provides memory functionality and enables temporal integration of optical stimuli, closely resembling key synaptic behaviors such as paired‐pulse facilitation and long‐term potentiation [21, 22, 23]. Importantly, the defect‐sensitive nature of κ‐Ga_2_O_3_ introduces reproducible device‐to‐device variations in photoconductive dynamics, even among devices fabricated under nominally identical conditions. Rather than being detrimental, this intrinsic variability can be harnessed as a physical source of uniqueness for hardware‐level authentication. Under identical ultraviolet excitation, each κ‐Ga_2_O_3_ PD generates a distinct and stable temporal photocurrent signature that reflects its microscopic defect signature. Such device‐specific response patterns offer a physically grounded foundation for hardware authentication, analogous to physical unclonable functions (PUFs), while eliminating the need for extra security circuitry or stored digital keys [24, 25, 26, 27, 28]. When combined with data‐driven classification methods, these temporal signatures can be efficiently recognized and distinguished at scale [29, 30, 31, 32, 33, 34].

At the same time, the PPC effect and multilevel conductance states accessible in κ‐Ga_2_O_3_ PDs suggest potential for neuromorphic inference beyond sensing and authentication. The gradual conductance modulation and long decay times inherent to PPC can be mapped to synaptic weight states, enabling hardware‐constrained implementations of artificial and spiking neural networks [35, 36]. In this regard, device‐aware algorithmic frameworks such as quantization‐aware training and spiking neural network models provide essential tools for evaluating how emerging optoelectronic synapses may support inference tasks under realistic hardware constraints. While full system‐level neuromorphic processors remain an important long‐term goal, such hardware‐aware simulations play a critical role in establishing feasibility and guiding future integration.

**FIGURE 1:**
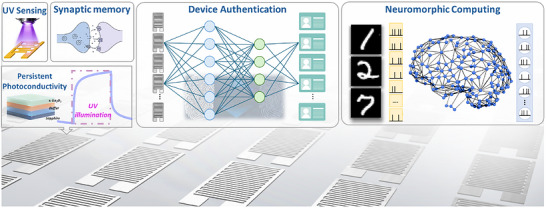
The device design concept, its structure, and PPC characterization. Schematic diagram of the functional classification of the device, including UV sensing, synaptic memory, device authentication and neuromorphic computing.

In this work, as shown in Figure 1, we report a multifunctional κ‐Ga_2_O_3_ based neuromorphic PD that unifies ultraviolet sensing, synaptic memory, hardware‐level authentication, and neuromorphic inference within a single device element. The κ‐Ga_2_O_3_ metal‐semiconductor‐metal (MSM) PDs exhibit distinct synaptic characteristics under pulsed ultraviolet illumination, including prominent paired pulse facilitation (PPF) and multi‐level conductance modulation. Specifically, robust photo synaptic behaviors are realized under stable 4 Hz pulsed light excitation, with a maximum photoresponsivity of 23.79 A W^−1^ achieved at an applied bias of 3 V. Moreover, the PPF index reaches as high as 116% under 260 nm UV illumination. To explore the feasibility of device‐level authentication, time‐series photoresponsivity data from different PDs are collected under identical pulsed UV illumination. A hybrid 1D deep embedding network (EmbedNet) is employed for hardware‐level authentication, achieving an area under the receiver operating characteristic curve (AUC) of about 0.97 and demonstrating robust cross‐cycle verification capability. To evaluate the potential of the proposed synaptic PDs for neuromorphic inference, a simulated spiking neural network (SNN) framework is constructed based on 16 conductance levels from the PD. A baseline fully connected artificial neural network (ANN) is first trained on the MNIST dataset and subsequently retrained using 4‐bit (16‐level) quantization aware training (QAT), where the quantized weights are mapped to the conductance states of κ‐Ga_2_O_3_ PDs [37]. The QAT‐trained model achieves a classification accuracy of 98.17%, surpassing its floating‐point counterpart and confirming the effectiveness of the quantization strategy. To incorporate temporal dynamics and align with neuromorphic hardware constraints, the quantized ANN is then converted into an SNN using leaky integrate‐and‐fire (LIF) neurons and surrogate gradient learning. The resulting SNN attains a classification accuracy of 96.80% on the same task, demonstrating minimal performance degradation despite quantization and spike‐based encoding.

Hence, by leveraging intrinsic device‐to‐device variability, time‐domain photocurrent responses are employed for hardware authentication using an EmbedNet, achieving high authentication accuracy without external cryptographic hardware. Furthermore, the neuromorphic inference capability of the devices is evaluated using a hardware‐aware simulation framework, in which experimentally extracted conductance states are mapped to QAT ANN and subsequently converted into SNN based on LIF neurons. By co‐localizing sensing, memory, authentication, and neuromorphic inference at the device level, this work establishes κ‐Ga_2_O_3_ synaptic photodetectors as a promising materials‐enabled building block for compact, intelligent, and privacy‐enhancing optoelectronic edge systems.

## 2 Methods

### 2.1 Material Growth and Device Fabrication

The O_2_ plasma MBE system (Riber) was utilized to grow the κ‐Ga_2_O_3_ film on the gallium nitride (GaN) /allium nitride (AlN)/c‐plane sapphire substrate. The base pressure in the chamber was 7 × 10^−10^ Torr. The optimized growth temperature was 650°C. Ga flux is supplied to a heated substrate by evaporating high‐purity molten Ga metal, and O radicals are produced by oxygen plasma for the growth of κ‐Ga_2_O_3_. As a measure of the metal flux, a pressure gauge at the sample position is used to determine the beam equivalent pressure (BEP). The plasma power during growth was kept constant at 480 W, and the O_2_ flow was set to 0.35 sccm. For our sample (κ‐Ga_2_O_3_), the BEP of Ga was 1.48× 10^−7^ Torr, and the temperature of the Sn cell was 340°C. Cross‐sectional scanning transmission electron microscopy (STEM) of as grown sample is shown in Figure S1.

The κ‐Ga_2_O_3_ sample was diced into 1×1 cm^2^ and cleaned with acetone and isopropyl alcohol (sonication for 10 min each). On the sample surface, a positive photoresist was spin‐coated. The sample was exposed to a direct writer (Heidelberg DWL 66+ laser lithography system) to make two interdigitated metal finger patterns on the κ‐Ga_2_O_3_ layer. After the development, the photodetector pattern is transferred onto the surface, and a 60 nm thick layer of titanium and a 120 nm thick layer of gold are deposited using reactive sputtering. Then, the photoresist was removed by acetone. For the implementation of the metal contact, the sample was rapidly annealed at 480°C for 1 min in N_2_ ambient.

### 2.2 Characterization

The surface morphology of the grown samples was examined using the scanning electron microscope (SEM, Thermo Scientific Helios 5 UX), atomic force microscopy (AFM, Bruker Dimension Icon SPM), and the structural properties were analyzed using X‐ray diffraction (HRXRD, Bruker D8 Discover) and transmission electron microscopy (TEM, Thermo Scientific Themis Z). The potential distributions of the samples were measured using the above AFM instrument under frequency‐modulated Kelvin probe force microscopy (FM‐KPFM) mode.

For the measurement of the fabricated photodetector's performance, deep ultraviolet (DUV) light were generated by a xenon‐mercury lamp system, passed through a monochromator (Oriel cornerstone CS260), dispersed by diffraction grating assembly (Newport 74060), and focused on the sample. The incident wavelength ranges from 200 nm to the visible wavelength range. Before the measurement, the light spectral density was calibrated by a commercial silicon photodetector (Newport 818‐UV), and neutral density (ND) filters controlled the intensity. The current‐voltage (I‐V) curves were measured by the semiconductor parameter analyzer (Agilent 4156C).

## 3 Results and Discussion

### 3.1 Synaptic κ‐Ga_2_O_3_ PDs Growth and Fabrication

**FIGURE 2:**
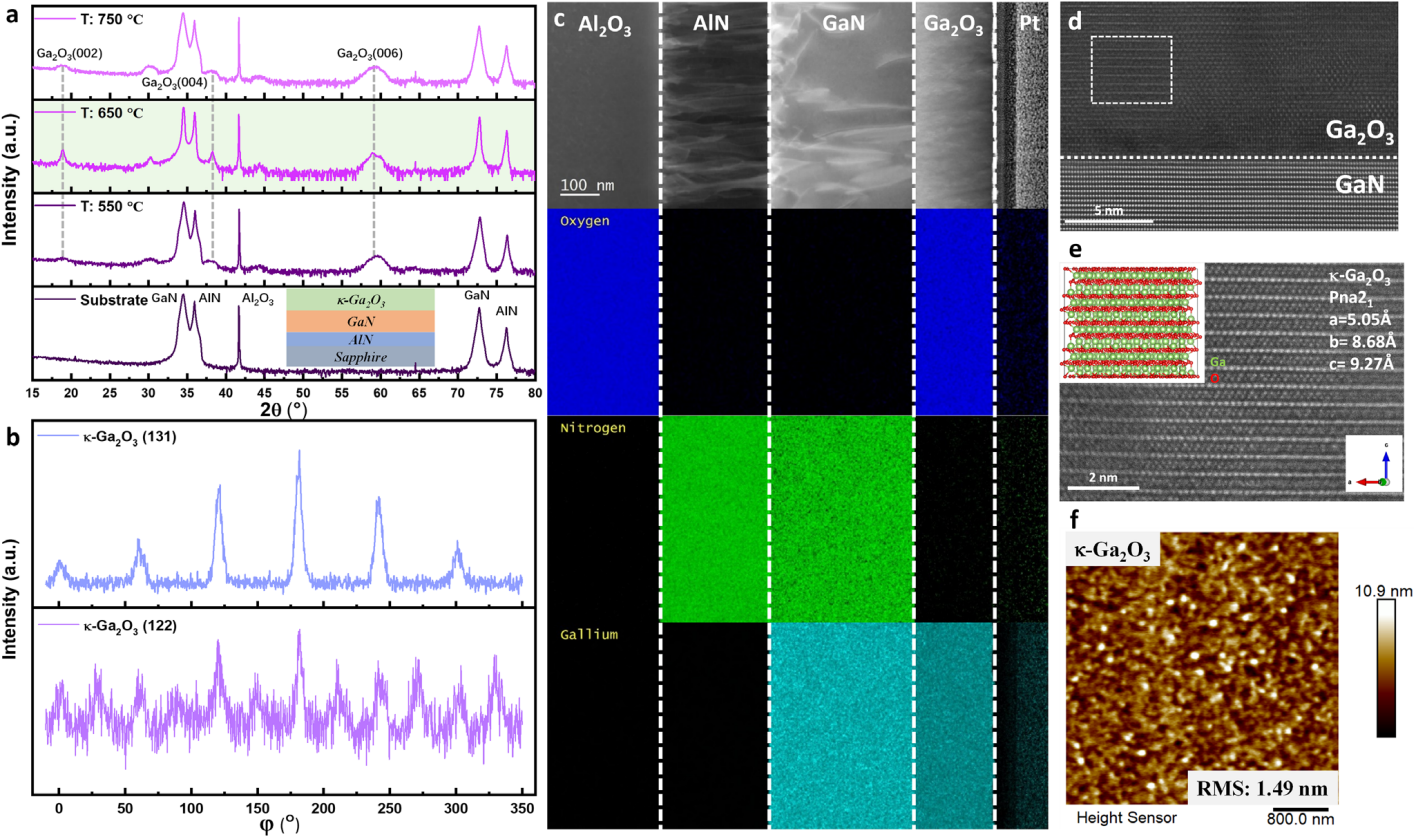
(a) XRD 2*θ*–*ω* scans of κ‐Ga_2_O_3_ thin film on GaN/AlN/Sapphire substrate under different growth temperatures and the GaN/AlN/sapphire substrate. The diagram of κ‐Ga_2_O_3_ sample growth structure is shown in the inset. (b) XRD φ‐scans of (131) and (122) of κ‐Ga_2_O_3_ thin film. (c) Cross‐sectional STEM image of the κ‐Ga_2_O_3_/GaN/AlN/sapphire sample and corresponding EDS mapping images of Al, Ga, and O elements. (d) STEM image of the κ‐Ga_2_O_3_/GaN interface. (e) The magnified view in (d) shows the atomic arrangement and the corresponding modeled crystal structure in the inset. (f) AFM image of the κ‐Ga_2_O_3_ thin film_._

The diagram of the as‐grown κ‐Ga_2_O_3_ sample growth structure is shown in the inset of Figure 2a. The AFM image (Figure 2f) shows the atomically flat features of the κ‐Ga_2_O_3_ sample with root mean square (RMS) roughness of 1.49 nm. This displays that the surface of the grown κ‐Ga_2_O_3_ epitaxial layer has a flat morphology, reaching a high level of film growth precision.

To evaluate the effect of growth temperature on the crystalline quality of κ‐Ga_2_O_3_ film, the XRD 2*θ*–*ω* scans were performed and shown in Figure 2a. For the substrate, characteristic diffraction peaks of GaN appear at 34.5° and 72.9°, corresponding to the (0002) and (0004) planes (ICDD PDF Card: 00‐050‐0792), while peaks of AlN are observed at 36° and 76.4°, assigned to the (0002) and (0004) planes (ICDD PDF Card: 00‐025‐1133). A diffraction peak from the Al_2_O_3_ (0006) plane is also observed at 41.66°. Compared to the substrate, the diffraction intensities of κ‐Ga_2_O_3_ exhibit a strong dependence on growth temperature. Samples prepared at 550°C, 650°C, and 750°C were systematically compared, with emphasis on the characteristic reflections of κ‐Ga_2_O_3_ (e.g., (002), (004), and (006) planes, which are commonly indexed at approximately 2θ values of 19.1°, 38.7°, and 59.7° (ICDD PDF Card: 04‐020‐4586), also, these peaks indicating preferential growth along the (001) direction as reported in all previous κ‐Ga_2_O_3_ growth literature [38].The XRD results confirm that the κ‐Ga_2_O_3_ sample grown at 650°C exhibits the most favorable crystalline characteristics, with the strongest peak intensity and narrowest full width at half maximum (FWHM) at (002) and (004) planes. To further confirm the structures, the additional Phi (φ) scan of κ‐Ga_2_O_3_ (131) and (122) reflections have been measured in Figure 2b. When 2*θ* value of 33.3° and Psi(ψ) position of 54.6° is performed, only the (122) reflection of the orthorhombic lattice can be found, as the hexagonal lattice has no reflections at this position. The result is shown at the bottom (the purple line). Therefore, the 12 peaks can be explained by three rotation domains of orthorhombic Ga_2_O_3_, each contributing four reflexes [39, 40]. In addition, cross‐sectional scanning transmission electron microscopy (STEM) and corresponding energy dispersive spectroscopy (EDS) mapping images clearly reveal the layered structure of κ‐Ga_2_O_3_/GaN/AlN/sapphire sample, as shown in Figure 2c. The Ga_2_O_3_ top layer appears uniform with sharp, abrupt interfaces to the underlying GaN buffer layer. No significant elemental interdiffusion is observed across any interface, confirming the good interface control during epitaxial growth. We further analyzed the nanostructures of the κ‐Ga_2_O_3_ film and the κ‐Ga_2_O_3_/GaN interface. The STEM shows the interface between the κ‐Ga_2_O_3_ and GaN substrate film in Figure 2d. The magnified STEM view in Figure 2e matches the modeled κ‐Ga_2_O_3_ structure in the figure inset. Collectively, these results demonstrate that, for the growth conditions applied here, κ‐Ga_2_O_3_ is in orthorhombic Pna2_1_ symmetry.

**FIGURE 3:**
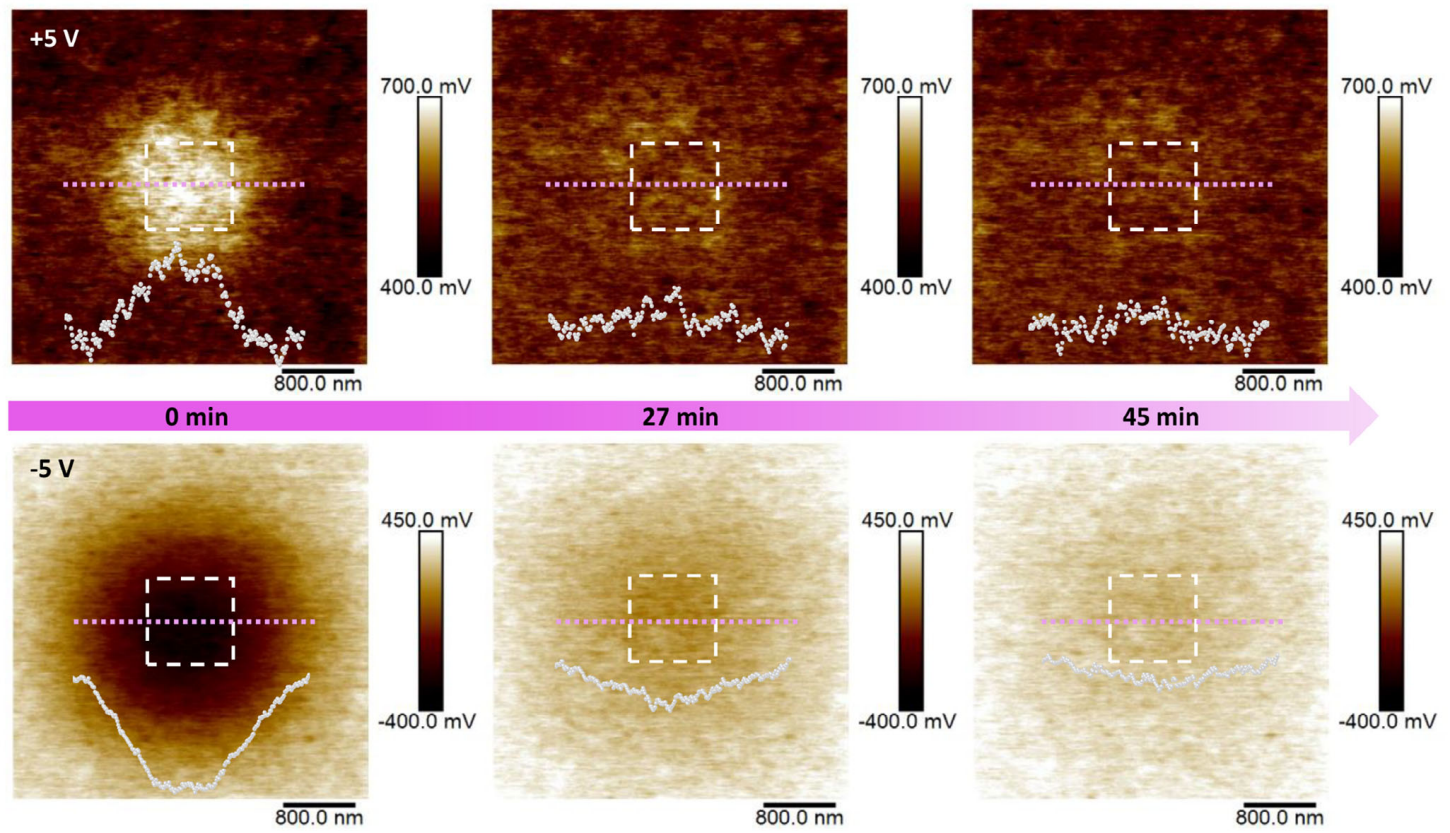
KPFM surface potential maps of κ‐Ga_2_O_3_ after hole injection (+5 V bias, top) and electron injection (−5 V bias, bottom). Insets show the corresponding potential profiles along the purple dashed lines.

Defects introduced during the material growth process, as well as those originating from intrinsic structural characteristics, commonly contribute to the PPC effect. In Ga_2_O_3_ based PDs, PPC effect, defined as the prolonged conductivity after the removal of illumination, is a commonly observed phenomenon [8]. However, point defects such as oxygen vacancies cannot be directly visualized by conventional TEM or STEM, due to their ultra‐small atomic‐scale size. In order to clearly validate the PPC characteristics of κ‐Ga_2_O_3_ thin films from material perspective, we performed Kelvin Probe Force Microscopy (KPFM) measurements to monitor the dynamic evolution of surface potential following charge injection [41]. As depicted in Figure 3, the κ‐Ga_2_O_3_ surface exhibits immediate and pronounced potential shifts after both positive and negative biasing: under +5 V bias (hole injection), a distinct positive potential elevation (from ≈ 443 to ≈721 mV, 0 min) is observed in the biased region, whereas a negative potential depression (from ‐290 to ≈320 mV, 0 min) occurs under −5 V bias (electron injection). These potential variations arise from the accumulation of injected carriers trapped in the deep defect levels within the κ‐Ga_2_O_3_, which disrupt the intrinsic surface charge equilibrium. Notably, the perturbed surface potential does not remain static but undergoes gradual relaxation toward the initial state over a timescale of tens of minutes. A closer comparison of the relaxation dynamics reveals similar temporal trends but subtle differences in the magnitude of potential recovery between holes and electrons: the bright high‐potential region induced by hole injection fades over the 45 min observation window, with a continuous decrease in the corresponding potential profile peak height, whereas the dark low‐potential region from electron injection brightens, and the profile valley depth diminishes. This disparity implies an asymmetry in the trapping/detrapping efficiency of κ‐Ga_2_O_3_ toward holes and electrons, which likely stems from the distinct energy levels and capture cross‐sections of deep defects for holes versus electrons. This characteristic may be exploited to tailor the synaptic plasticity of κ‐Ga_2_O_3_‐based neuromorphic devices. Overall, this dynamic relaxation directly reflects the trapping and detrapping kinetics of carriers in the deep defects; the slow recovery rate (tens of minutes) indicates that the captured carriers exhibit prolonged lifetimes in the defect states, which constitutes the core physical origin of the PPC effect in κ‐Ga_2_O_3_.

Furthermore, the symmetric relaxation trends for both hole and electron injection, with complete recovery to the initial potential, confirm the reversibility of charge trapping and detrapping in the deep defects. This reversible charge storage behavior is critical for neuromorphic applications, as it enables the reconfigurability of synaptic weights. Additionally, the spatially uniform potential distribution across the biased region suggests a homogeneous distribution of deep defects in the κ‐Ga_2_O_3_ film, which is essential for ensuring the consistency of device performance. Collectively, these KPFM results provide compelling experimental evidence for the intrinsic PPC effect of κ‐Ga_2_O_3_, and demonstrate its potential for developing high‐performance neuromorphic devices.

**FIGURE 4:**
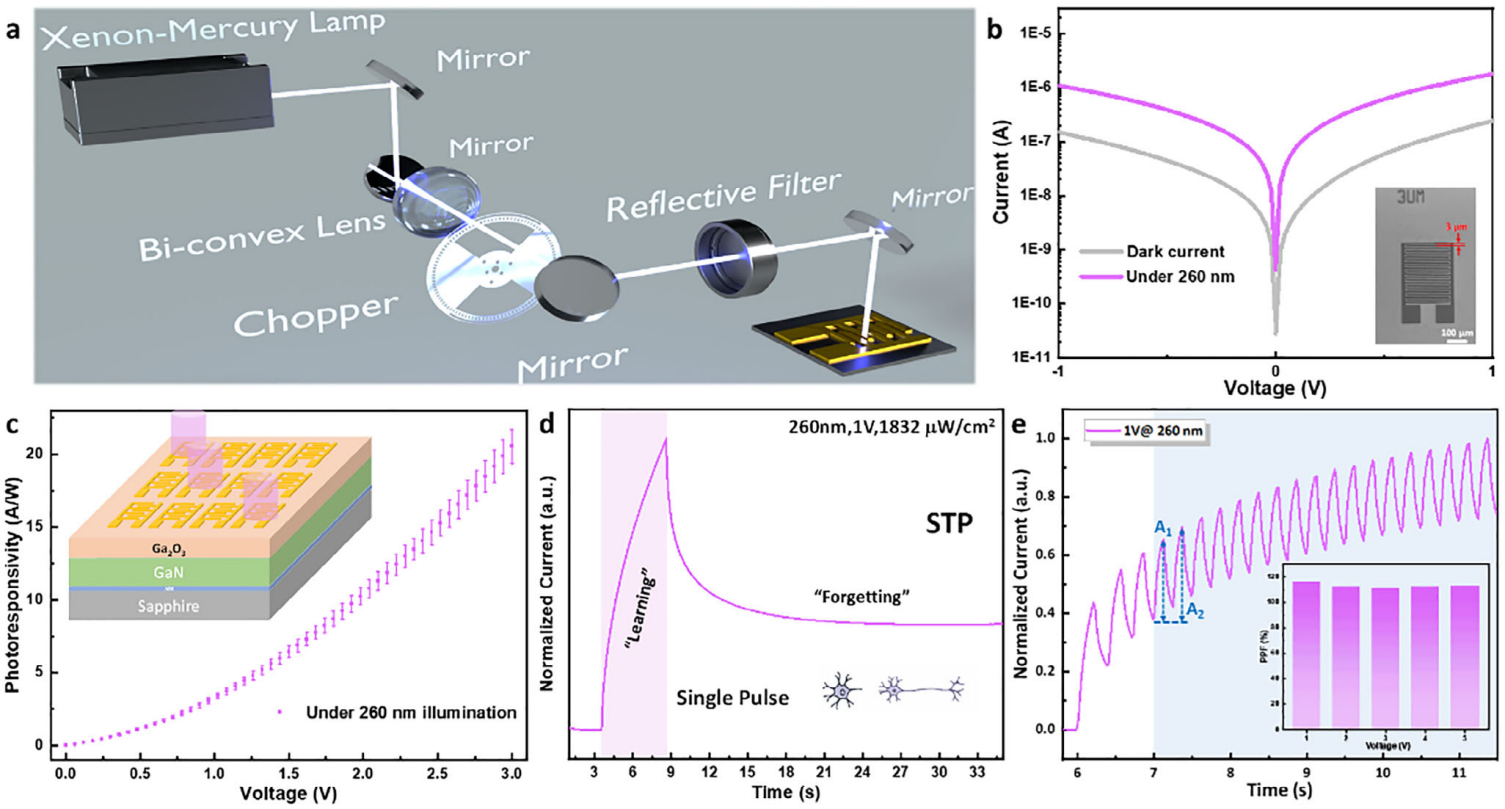
(a) The diagram of measurement system setup. (b) *I*–*V* characteristics curve of the κ‐Ga_2_O_3_‐based photodetector under dark and 260 nm illumination, respectively, with an inset showing the SEM image of the device. (c) Photoresponsivity variations as a function of applied voltage, a schematic diagram of the fabricated κ‐Ga_2_O_3_‐based photodetector is shown in the inset. (d) The photocurrent was induced by a single 260 nm spike with a duration of 5 s and power density of 1832 µW cm^−2^. (e) The photoresponse is triggered by light pulses at a stable frequency of 4 Hz, under applied voltages at 1 V. The PPF index remains stable under different applied voltages, with values ranging between 110% and 116% is shown in inset.

Then, the diagram of the fabricated device is shown in Figure 4c inset. Figure 4b shows current–voltage (*I*–*V*) curves of the fabricated device measured under dark and UVC illumination (*λ* ≈ 260 nm, *P* ≈ 110 µW). At 1 V, the off‐current (*I*_off_) and on‐current (*I*_on_) were 0.24 and 1.81 µA, respectively. The high dark current can be explained by the symmetric metal design, which will be optimized by using different metal contacts and will be investigated in the future. Figure 4c shows voltage‐dependent photoresponsivity variations of the 5 devices from 0–3 V with a 60 mV step. The highest responsivity can reach 23.79 A W^−1^ at 3 V. Next, we systematically study the performance of the devices as photo synapses based on the PPC effect. The UV light is used to simulate external stimuli, and the device can convert the light signal into the photocurrent, corresponding to the neurotransmitters between the biological synapses [42]. Similar to the learning process in the human brain, repeated stimulation induces a longer‐lasting and stronger memory effect. Compared to the response under a single light pulse in Figure 4d, continuous light pulses with a stable frequency, as shown in Figure 4e, generate a higher photocurrent and require more time to return to the initial state. This implies that increased memory levels and the transition from short‐term plasticity (STP) to long‐term plasticity (LTP) can be achieved through external stimulation, such as increasing the light pulse width or the number of pulses. Thus, the device can successfully simulate the complete learning to forgetting cycle of biological synapses.

To investigate the time‐dependent behavior of the photoresponse and collect the photoelectric raw data for device authentication and devicconductance value extraction, we used the same setup shown in Figure 4a, where we integrated an optical chopper with continuous light pulses at a stable frequency of 4 Hz. Under this ongoing stimulation, the photocurrent continued to increase with the increasing injection time, demonstrating the accumulation of generated photo‐carriers due to the PPC effect in Ga_2_O_3_, just like the memory behavior of synapses. The PPC refers to a prolonged photoconductive response that persists after the cessation of light exposure. Paired‐pulse facilitation (PPF) reflects synapse‐like behavior in which the response to the second light pulse (A_2_) is enhanced compared to the first pulse (A_1_). PPC extends the carrier recombination time, leaving residual photo‐generated carriers or trapped states after the first pulse. These residual carriers enhance the response of the second pulse (A_2_), thereby increasing the PPF index. Therefore, we use the PPF index (PPF index = A_2_/A_1_) to quantify the PPC effect as shown in Figure 4e. Our previous research explored the correlation between the PPC and PPF in Ga_2_O‐based photodetectors [43]. PPC prolongs carrier recombination, enhancing the PPF effect. Based on the photocurrent values under continuous light pulses, the inset in Figure 4e summarizes the PPF index under the same 260 nm light illumination. The PPF index remains stable under different applied voltages (1‐5 V), with values ranging between 110% and 116%. The results demonstrate that the κ‐Ga_2_O_3_‐based photodetector exhibits a strong persistent PPC effect even under a low applied voltage. Therefore, an operation voltage of 1 V was selected for subsequent device authentication and neural network input data acquisition to ensure low energy consumption. Our structure highlights the dual functionality of the synaptic device, encompassing both hardware‐level authentication and neuromorphic computing, which will be discussed in the following sections.

### 3.2 Device‐Level Authentication

**FIGURE 5:**
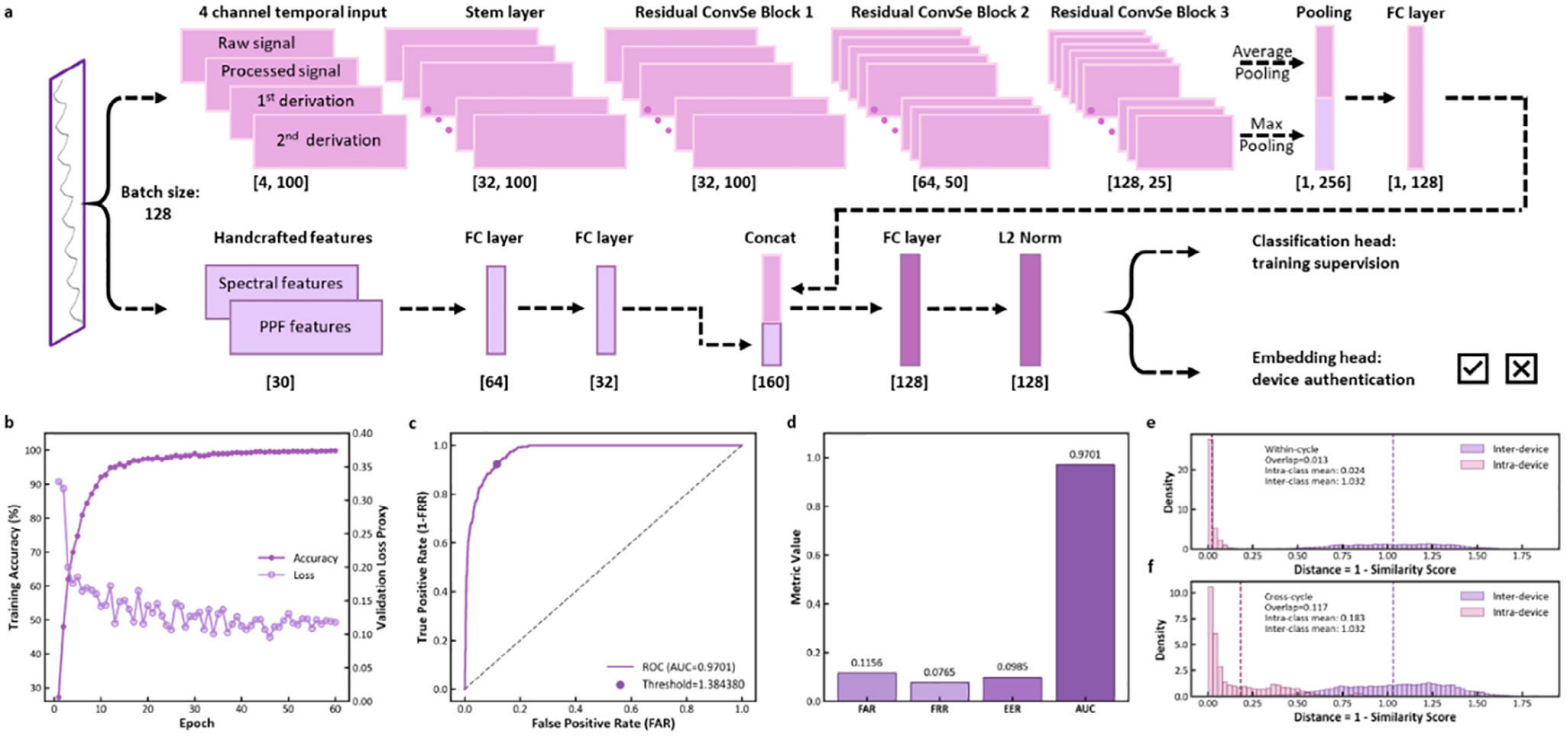
EmbedNet and authentication performance of the neuromorphic photodetector. (a) Architecture of the hybrid EmbedNet integrating temporal convolutional features and handcrafted physical descriptors. (b) Training accuracy and loss during network optimization. (c) ROC curve of the authentication system. (d) Summary of authentication metrics including FAR, FRR, EER, and AUC. (e) Distributions of similarity distances for intra‐device and inter‐device samples within the same measurement cycle. (f) Corresponding similarity distance distributions under cross‐cycle evaluation.

Figure 5a illustrates the architecture of the proposed hybrid EmbedNet designed for device authentication using temporal response signatures from the neuromorphic photodetector. Each input sample consists of a 100‐point temporal segment extracted from the device response. To preserve both waveform morphology and dynamic variations, four synchronized temporal channels are constructed, including the normalized raw signal, the preprocessed signal, and the first‐ and second‐order temporal derivatives. This four‐channel representation captures both the global temporal profile and local transition dynamics of the device response.

The temporal feature extraction branch begins with a 1D convolution‐batch normalization‐Gaussian error linear unit (Conv1D‐BatchNorm‐GELU) stem layer that expands the input channels to 32 feature maps. The network then passes through three residual convolutional squeeze‐and‐excitation (ConvSE) blocks [44], which progressively learn hierarchical temporal representations while adaptively reweighting informative channels. The feature dimensionality increases from 32 to 64 and finally to 128 channels while the temporal resolution is gradually reduced through strided convolution. Global average pooling (GAP) and global max pooling (GMP) are then applied and concatenated to obtain a compact 256‐D representation of the temporal signal. In parallel, a lightweight branch processes handcrafted physical descriptors derived from the device dynamics. These descriptors include PPC kinetic features including amplitude range, rise and decay characteristics, slope statistics, and autocorrelation, as well as spectral descriptors obtained from the fast Fourier transform (FFT) of both the raw and processed signals. These handcrafted features capture interpretable physical characteristics of the device dynamics and complement the convolutional representations learned from the temporal waveform. The handcrafted descriptors are processed through two fully connected (FC) layers to produce a 32‐D feature embedding. The temporal representation and handcrafted features are then concatenated to form a joint representation, which is further projected into a 128‐D embedding space followed by L2 normalization. During training, an auxiliary classification head is used to predict the device identity and provide discriminative supervision. The network is optimized using a combination of cross‐entropy loss (CE) and supervised contrastive loss (SupCon), which encourages embeddings from the same device to cluster together while separating those from different devices. To evaluate robustness against temporal variability, the dataset is divided following a strict cross‐cycle protocol. For each device, three measurement cycles are used for training, one cycle is used for validation, and another independent cycle is used for testing. This ensures that the model learns device‐intrinsic fingerprints rather than memorizing specific measurement instances.

For authentication, the embedding vectors generated by the network serve as device fingerprints. A query embedding is compared with device prototypes using similarity scoring, and authentication decisions are made by thresholding the similarity value, consistent with the verification mechanism of PUF systems. The training behavior of the proposed network is shown in Figure 5b, where the training accuracy rapidly converges to approximately 100% while the loss decreases steadily, indicating stable learning of device‐specific temporal signatures. The authentication performance is further evaluated using the Receiver Operating Characteristic (ROC) curve shown in Figure 5c. The curve demonstrates a strong discrimination capability between genuine and impostor responses, achieving an AUC of approximately 0.97. Key authentication metrics derived from the ROC analysis are summarized in Figure 5d, including the false acceptance rate (FAR), false rejection rate (FRR), EER, and AUC. Specifically, the FAR is 0.1156, indicating that approximately 11.56% of impostor attempts are incorrectly accepted as genuine devices at the selected decision threshold. The FRR is 0.0765, meaning that 7.65% of genuine device responses are mistakenly rejected during authentication. The corresponding EER is 0.0985, representing the operating point where FAR and FRR become equal. In PUF‐based authentication systems, a lower EER indicates better overall discriminability between genuine and impostor responses. Together, these results demonstrate that the proposed hybrid embedding network effectively captures device‐specific temporal fingerprints and enables reliable device authentication. Figure 5e shows the distributions of similarity distances for intra‐device and inter‐device samples within the same measurement cycle. Figure 5f further evaluates the distributions under cross‐cycle conditions, where the training and testing samples originate from different measurement cycles. Compared to overlap value within the same cycle, the overlap between intra‐device and inter‐device distributions increases to 0.117, reflecting additional variability introduced by cycle‐to‐cycle fluctuations in the device response. Nevertheless, the two distributions remain well separated in terms of their statistical centers: the mean intra‐device distance is 0.183, whereas the mean inter‐device distance is 1.032, which is nearly six times larger. This large separation ensures that device signatures remain distinguishable despite the increased overlap. In practical authentication scenarios, multiple temporal segments from the same device are aggregated during verification. Such multi‐segment averaging effectively reduces random fluctuations in individual measurements and further enlarges the statistical margin between intra‐device and inter‐device similarities, thereby enabling reliable device authentication. These results collectively indicate that the proposed architecture successfully extracts robust device‐specific embeddings suitable for PUF‐based authentication.

### 3.3 Hardware‐Aware Simulation Framework Towards Neuromorphic Computing

After verifying the effectiveness of the κ‐Ga_2_O_3_ PD in device‐level authentication, we further investigated its potential in neuromorphic computing. While the previous method focused on identity recognition based on signal variability, it treats the photodetector primarily as a passive input source. In contrast, the device's physical behavior, including persistent photoconductivity and conductance modulation under light stimulation, indicates that it can work as an artificial synapse and participate directly in neural computation. To identify an appropriate excitation wavelength, we performed pulse response measurements over a wavelength range from 230 to 290 nm. Figure S2 depicts the photocurrent response of the device under light pulses of different wavelengths, verifying that the device exhibits distinct and wavelength‐dependent photoresponse behavior. To achieve a wide, linear, and well‐resolved multi‐level conductance range, we selected 260 nm as the optimal excitation wavelength for the subsequent synaptic measurements. When exposed to 1 to 16 consecutive pulses, the conductance of a single device increased from 4.7834×10^−6^ to 5.4121×10^−6^ S in clearly distinguishable steps. This forms a set of 16 stable and repeatable conductance levels, suggesting the feasibility of multi‐bit weight storage at the device level. In neural network implementations, weights with linear behavior are typically more desirable.

**FIGURE 6:**
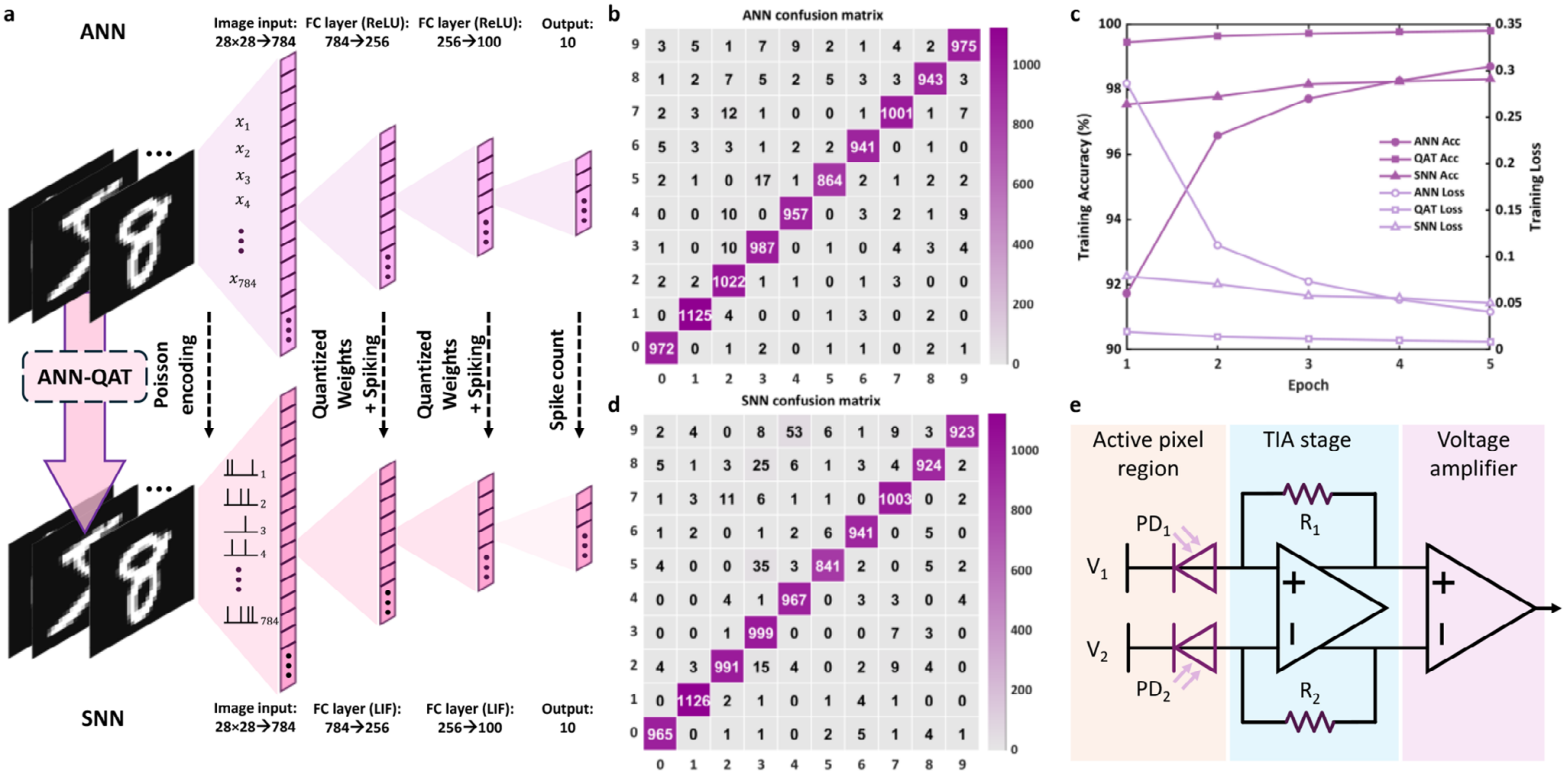
(a) The architecture of the ANN and SNN. (b) Confusion matrix of ANN. (c) Confusion matrix of SNN. (d) Training accuracy and loss over 5 epochs. (e) Schematic of the differential photodiode readout architecture.

To evaluate its practical applicability, a fully connected neural network is constructed first for MNIST handwritten digits classification as shown in Figure 6a [45]. The network consists of an input layer with 784 neurons, two hidden layers with 256 and 100 neurons, and an output layer of 10 neurons. It is first trained as a standard ANN, achieving a baseline accuracy of 97.87%. To reflect the discrete nature of conductance in the physical device, QAT is applied during network training. The weight values are constrained to 16 levels, corresponding to the 16 conductance states observed experimentally. The QAT‐trained model achieves 98.17% accuracy, slightly surpassing the baseline ANN. This confirms that the network maintains high performance under discrete weight constraints. To align with the event‐driven characteristics of neuromorphic hardware, the quantized ANN is converted into a SNN. The conversion uses a LIF neuron model with surrogate gradient training. The quantized weights are directly mapped to the SNN, which processes spikes over 16 discrete time steps. The final classification result is determined by accumulating output spikes during this time window. The SNN achieves 96.80% accuracy on the same task. The confusion matrix of the ANN and SNN for test set are shown in Figure 6b,c, separately, which possess similar accuracy. Figure 6d shows the training accuracy and loss of three different neural networks in the MNIST dataset. Based on the trained weights from ANN and QAT, SNN model possesses a high training accuracy at the beginning, showing minimal performance degradation compared to the ANN model. These results indicate that the experimentally measured conductance states of the κ‐Ga_2_O_3_ PD can be mapped to synaptic weights and effectively used for simulation‐based neural network training, demonstrating the feasibility of device‐informed neuromorphic computing.

Due to the MSM structure adopted for the κ‐Ga_2_O_3_ PD in this work, the device conductance cannot be reduced by applying a forward bias, which precludes conventional bidirectional weight modulation within a single device. To address this limitation at the architectural level, a differential readout scheme is proposed, as shown in Figure 6e, in which two PDs are paired to form the G^+^ and G^−^ branches of a single synapse. The active pixel region consists of reverse‐biased PDs (PD_1_ and PD_2_) subjected to optical illumination. Their photocurrents are fed into a differential transimpedance amplifier (TIA), where the current difference is converted into a differential voltage signal. A subsequent voltage amplification stage further processes the differential output. This configuration enables effective bidirectional weight representation using only unidirectional, light‐induced programming, while allowing finer granularity through an increased number of accessible conductance states. In the neuromorphic computing mode, the differential architecture supports multi‐level, tunable synaptic weights with expanded inter‐weight distances, which mitigates weight clustering and enhances the representational granularity of synaptic states [46, 47]. In contrast, when operating as a PUF source, the use of balanced PD pairs suppresses noise‐induced variations at the readout level, thereby improving sensitivity to intrinsic device‐level differences. This results in an enhanced separation between extracted features from different devices, enabling more reliable authenticity verification.

Extending this design to an array configuration further enlarges the challenge response space, strengthening the entropy and robustness of the PUF. Overall, the proposed differential architecture provides a unified hardware primitive that supports both programmable synaptic weighting and robust device authentication, laying the groundwork for future implementations of privacy‐preserving, on‐chip spiking neural network learning.

## 4 Conclusion

In summary, we present a comprehensive investigation of κ‐Ga_2_O_3_‐based PDs as multifunctional photo‐synaptic devices for intelligent optoelectronic systems, encompassing thin film growth, device fabrication, optoelectronic characterization, AI‐assisted extraction of device‐specific features for hardware authentication, and hardware‐aware evaluation of neuromorphic inference. The κ‐Ga_2_O_3_ MSM PDs exhibit pronounced light and bias controlled synaptic behaviors, achieving a peak photoresponsivity of 23.79 A W^−1^ at 3 V under 260 nm illumination and a PPF index of 116% under the same conditions. Leveraging the intrinsic defect‐mediated variability in temporal photocurrent dynamics, device‐specific response signatures are exploited as physical entropy sources for hardware‐level authentication and are effectively resolved by a hybrid 1D EmbedNet, yielding an AUC of about 0.97 under cross‐cycle evaluation. In parallel, the multi‐level, optically tunable conductance states of the PDs are mapped to analog synaptic weights and evaluated within a hardware‐aware SNN framework. By employing QAT and LIF neuron models, MNIST handwritten digit recognition is achieved with high inference accuracy and limited performance degradation under device‐constrained operation. By co‐localizing sensing, memory, authentication, and inference within a single wide‐bandgap material platform, this work establishes κ‐Ga_2_O_3_‐based synaptic photodetectors as a promising building block for compact, intelligent, and privacy‐enhancing optoelectronic hardware targeting next‐generation edge intelligence applications.

## Conflicts of Interest

The authors declare no conflicts of interest.

## Supporting information




**Supporting File**: advs75160‐sup‐0001‐SuppMat.docx.

## Data Availability

The data that supports the findings of this study are available from the corresponding author upon reasonable request.
