# Supplementary material for: Synaptic κ‐Ga2O3 Photodetectors for Privacy‐Enhancing Neuromorphic Computing
Source: Adv Sci (Weinh). 2026 Apr 3;13(36):e75160. doi: 10.1002/advs.75160 (PMC13317656; doi:10.1002/advs.75160)
Supplement: Supplementary file 1 — Supporting File: advs75160‐sup‐0001‐SuppMat.docx. [file ADVS-13-e75160-s001.docx]

Supporting Information

**Synaptic κ-Ga_2_O_3_ Photodetectors for Privacy-Enhancing Neuromorphic Computing**

*Yanqing Jia, Heming Lin, Hongliang Chang, Wenqing Niu, Yue Wang, Hang Lu, Abdullah AlQuwayzani, Yara Banda, Long Chen, Qingxiao Wang, Bambar Davaasuren, Mohamed Ben Hassine, Tien Khee Ng***and Boon S. Ooi**

Fig S1: Cross-sectional scanning transmission electron microscopy (STEM) of as grown sample.

Fig S2: Photocurrent characteristics of the device under light pulses of different wavelengths.
